# Social determinants of seeking emergency and routine dental care in Saudi Arabia during the COVID-19 pandemic

**DOI:** 10.1186/s12903-021-01577-1

**Published:** 2021-04-26

**Authors:** Dalia E. Meisha, Ahad Mosallem Alsolami, Ghaliah Muslih Alharbi

**Affiliations:** 1 Department of Dental Public Health, Faculty of Dentistry, King Abdulaziz University, P.O. Box 80209, Jeddah, 21589 Saudi Arabia; 2 Ministry of Health, Tabuk, Saudi Arabia; 3 Ministry of Health, Madinah, Saudi Arabia

**Keywords:** COVID-19, Health-seeking behavior, Dental care, Teledentistry, Saudi Arabia, Pandemic, SARS-CoV-2, Survey

## Abstract

**Background:**

Between March and June 2020, closing dental clinics during the COVID-19 pandemic except for emergency dental care was recommended. It is documented that health-seeking behaviors change during pandemics. The objective of this study was to examine social determinants associated with decisions to seek dental care in Saudi Arabia during the COVID-19 pandemic.

**Methods:**

A total of 4372 participants were invited to this cross-sectional web-based survey distributed from April 21 to June 20, 2020. The survey included a list of emergency, urgent, and routine dental procedures. Participants were asked if they would seek dental care for these conditions during the pandemic, and what pain severity would make them seek dental treatment. Logistic regression models were performed for predicting variables that explain the decision to go or not to go to the dental clinic during the pandemic for each dental condition.

**Results:**

A total of 3443 responded to this survey. The emergency dental situation participants were most willing to go to the dental clinic for was trauma involving facial bones compromising the airway (94.5%). Only 65.8% were willing to seek care for facial cellulitis compromising the airway. On average 35.2% reported seeking teleconsultation as the first step. Eighteen percent of participants were still willing to go to the dental clinic during the pandemic for routine dental procedures. Multiple logistic regression showed that females (Odds Ratio (OR): 1.6, OR 95% CI 1.3, 1.9), people who had never visited a dentist (OR: 1.8, OR 95% CI 1.3, 2.5), and people living in metropolitan regions (OR: 1.8, OR 95%: 1.4, 2.3) had higher odds for not seeking emergency dental care during this pandemic. The pain threshold for seeking dental care during the pandemic was 7 out of 10. Female, those who never visited a dentist, and those from urban regions reported higher pain threshold before seeking dental care (*P* value < 0.001).

**Conclusion:**

Social disparities were found in emergency dental care seeking decision-making in Saudi Arabia during the COVID-19 pandemic. It was alarming that some people were afraid to seek dental care for life-threatening dental emergencies as cellulitis during this pandemic. This reflects the importance of increasing public health awareness and governmental regulations.

**Supplementary Information:**

The online version contains supplementary material available at 10.1186/s12903-021-01577-1.

## Background

On January 30, 2020, the World Health Organization (WHO) declared the novel coronavirus outbreak as a “global public health emergency of international concern” [1]. Later on February 11th, the official name severe acute respiratory syndrome coronavirus 2 (SARS-CoV-2) was announced, also known as COVID-19 [2]. On March 2, 2020, the first Coronavirus Disease 2019 (COVID-19) case was confirmed in the Kingdom of Saudi Arabia (KSA) [3]. Soon after, the Saudi Ministry of Health (MOH) initiated campaigns to increase the public awareness about the routes of COVID-19 transmission and prevention methods. This was followed by strict measures such as curfew or lockdown to control transmission at the national level. KSA has 13 administrative regions, some of which applied 24-h lockdown, while others applied partial lockdown based on the number of active/critical/death cases and the burden on the healthcare system. In addition, places with high risk of transmission such as shopping malls, restaurants, coffee shops and barbershops were closed until June 20, 2020. During this period, telemedicine was activated through the universal MOH hotline (937). In the same period, dental clinics were advised to close except for dental emergencies per the MOH recommendations [4]. This recommendation was aligned with that of the United States Centers for Disease Control and Prevention (CDC), the American Dental Association (ADA) [5] and other international dental organizations [6]. Furthermore, postponing non-urgent dental procedures was recommended, and dental patients were advised to contact their dental practices via teleconsultation in order to triage cases based on urgency and plan management accordingly [7, 8].

Dental patients and staff are at risk of cross-infection due to the nature of dental settings [9]. Moreover, most dental procedures are aerosol-producing, especially those using the dental hand piece [10]. This poses a risk of spreading infection in the dental office during the pandemic [11, 12]. It has been documented that the virus in the aerosol can stay viable for more than 3 h with high surface stability exceeding 72 h [13]. It was evident that aerosol-generating procedures were avoided for urgent dental care during the first six weeks of the COVID-19 pandemic in the UK, as they accounted for only 0.8% of all procedures. Three-fourth of patients undergoing triage needed clinical consultation with the most common reasons for urgent dental care being symptomatic irreversible pulpitis and symptomatic apical periodontitis, followed by acute dental abscess. The most common treatment provided was extraction (63%), followed by antibiotic prescription (13%) [14]. Another study found that the incidence of trauma cases in emergency departments during COVID-19 decreased with the majority being described as minor soft tissue trauma [15]. A Chinese study observed a 38% decrease in dental emergency patients and a 13% decrease in non-urgencies after the announcement of the COVID-19 pandemic. Also the types of conditions for which urgent dental treatment is sought has changed, as the proportion of orofacial infections increased by 20.9%, while dental trauma conditions decreased by 3.7% [16].

Few published studies explored dental patients’ attitudes regarding dental care visits during previous outbreaks. Yip et al. explored such behaviors during the 2003 severe acute respiratory syndrome (SARS) outbreak in Hong Kong. They found that about two-thirds of dental patients were afraid of contracting SARS during dental treatment, and therefore would avoid dental treatment [17]. Another study assessed dental patients’ apprehension during the Middle East Respiratory Syndrome (MERS) outbreak in 2014 in Riyadh, KSA. It was reported that 26% of participants expressed apprehension about undergoing dental care due to fear of contracting the infection [18]. A recent study reported that about 84% of pediatric dental patients’ parents from China would not take their child for dental care, even in the urgent case of severe toothache. Moreover, 92% of parents believed that a dental clinic is a place with high risk of virus transmission [19].

Health-seeking behavior has been reported to change during pandemics [20, 21]. In 2015, it was found that health-seeking behaviors were affected during the Ebola epidemic in Sierra Leone. This was demonstrated in the form of underutilization of health services and an increase in self-medication [20]. Another study reported the dominance of seeking help from outside of health facilities during outbreaks, which highlights the importance of public health education [21]. During the latest COVID-19 pandemic in China, the majority of patients with acute respiratory infections refrained from seeking medical assistance [22]. This reflects that further understanding of the health-seeking behavior for dental care during the COVID-19 pandemic is needed. The objective of this study was to examine the social determinants associated with the decision to seek dental care in Saudi Arabia during the COVID-19 pandemic.

## Methods

### Study design and participants

A cross-sectional survey was conducted, targeting people living in KSA during the period the Saudi authorities declared COVID-19 pandemic restrictions and imposed partial and/or complete lockdown. The survey was distributed during the lockdown period occurring from April 21 to June 20, 2020. The Research Ethics Committee at the Faculty of Dentistry, King Abdulaziz University (#035-04-20) approved the study. A non-probability snowball sampling technique was used to recruit participants. Inclusion criteria for participants were that they live in KSA and be age of 18 years or older. Workers in the dental field were excluded.

A web-based, self-administered, anonymous survey using the QuestionPro platform was used to collect responses electronically during the COVID-19 lockdown period. An electronic letter of invitation was embedded in the survey link. Once a person received the survey and clicked on the survey link, the QuestionPro platform counts this person as a recipient of the survey. Recruitment was solicited through the social media platforms commonly used in Saudi Arabia including WhatsApp, Twitter, Instagram, and Snapchat. The survey was distributed on twitter, Instagram, and Snapchat using regional news accounts, while distribution through WhatsApp used snowball sampling. The survey was made available in both Arabic and English languages.

### Survey instrument

The survey instrument consisted of five sections (Additional file 1: Survey). The first section collected sociodemographics. The second section asked about the details of the participant’s last dental visit (when, where, and why). The third section included a list of emergency and urgent dental situations that may require dental care during COVID-19 pandemic. This list was obtained from the ADA list of dental emergencies during the COVID-19 pandemic [23] and Ministry of Health (MOH) guidelines [4]. Both lists were similar except that the MOH guidelines had four additional urgent dental situations: an ulcer persisting for more than 3 weeks, dentin sensitivity, temporomandibular joint (TMJ) arthralgia and/or myalgia, and broken bonded retainers. The ADA makes a distinction between emergency and urgent dental conditions by defining dental emergencies as “potentially life-threatening and require immediate treatment including uncontrolled bleeding, cellulitis potentially compromising the patient’s airway, and facial bones trauma potentially compromising the patient’s airway” and defining urgent dental conditions as “conditions that requires immediate attention to relieve severe pain and/or risk of infection” [23]. Participants were asked “What would you do if you had such a dental condition now?” The responses to emergency and urgent dental situations were grouped into three categories: The first category was choosing to go to the dental clinic, the second category was using teleconsultation and subsequently going to the dental clinic if advised to do so, and the third category was choosing not to go, even if advised to do so. Photographic images were included for the cellulitis and localized abscess questions to illustrate the situation to lay people. The fourth section included a list of routine non-urgent dental care procedures. Lastly, the participants were asked to identify the severity of pain that would make them seek dental treatment during the pandemic using the Numeric Rating Scale (NRS-11) in which 0 represents no pain and 10 represents the worst pain possible [24]. Informed consent was obtained from participants who agreed to contribute to this study. Content validity of the survey was ensured by aligning it with the ADA list of dental emergencies [23]. This was further assessed through a panel of dental specialists and general dentists. Furthermore, face validity was checked with 20 laypeople that are not from a health-related discipline. Pilot testing was performed for both language versions before instigation of the recruitment, and further adjustments were made accordingly.

### Study variables

The social determinants considered included sociodemographic data and characteristics of the last dental visit. Gender, age, nationality, education level, employment status, marital status, income, KSA region, and characteristics of the last dental visit were considered as independent variables. KSA regions were categorized according to the population as follows: Metropolitan regions with population of more than 4 million (Riyadh, Makkah, Eastern province), regions with population between 1 and 4 million (Madinah, Qassim, Asir, Jazan), and regions with population less than 1 million (Najran, Aljouf, Albaha, Hail, Tabouk, Northern border). Participants’ decision to go or not to go to the dental clinic for each dental condition was considered to be the dependent variable.

### Sample size

Sample size was calculated using G-power (Version 3.1.9.3). The minimum sample size to conduct this study is 1536 with at least 384 per KSA region stratum, assuming a 5% margin of error and 95% confidence level. The web-based survey was closed on the last day of the lockdown.

### Statistical analyses

Statistical analyses were performed using IBM SPSS Statistics (Version 24, Armonk, NY: IBM Corp). Bivariate analyses were performed using Chi-square, Mann Whitney tests or Kruskal–Wallis tests, as appropriate. Logistic regression models were performed for predicting variables that explain the decision to go or not to go to the dental clinic during the pandemic for each dental condition. Significance level was established at 0.05.

## Results

### Sociodemographic characteristics

Of the 4372 who received the survey, 208 were ineligible, and 3443 completed the survey, which corresponds to a response rate of 78.7%. Reasons for exclusion were living outside KSA (n = 77) and being under the age of 18 years old (n = 131). The demographic characteristics are shown in Table 1. The majority of the participants (95.9%) completed the Arabic version of the survey, and it took 9 min on average to complete it. About 63% of respondents were female. Completion of the bachelor’s degree was the most frequently reported education level (61.5%). The majority of participants (77.3%) reported an income of SR20,000 or less. All KSA administrative regions were represented.

**Table 1:** Sociodemographic characteristics of survey participants from a sample of KSA population (n = 3443)

Characteristics	Male n = 1274		Female n = 2169		Total n = 3443	
	%	N	%	n	%	n
**Nationality**						
Saudi	93.5%	1191	94.1%	2041	93.9%	3232
Non-Saudi	6.5%	83	5.9%	128	6.1%	211
**Age**						
18–24 years	15.1%	192	25.4%	550	21.6%	742
25–29 years	14.1%	179	15.3%	331	14.8%	510
30–39 years	25.2%	321	23.1%	500	23.8%	821
40–49 years	21.6%	275	19.2%	416	20.1%	691
50–59 years	17.6%	224	10.6%	229	13.2%	453
60+ years	5.8%	74	2.7%	59	3.9%	133
**Marital status**						
Single	28.3%	361	40.1%	869	35.7%	1230
Married	69.9%	891	53.3%	1156	59.5%	2047
Divorced	1.4%	18	4.8%	104	3.5%	122
Widowed	0.3%	4	1.8%	40	1.3%	44
**Education level**						
Highschool or less	14.9%	190	19.4%	421	17.7%	611
Diploma degree	11.2%	143	3.2%	70	6.2%	213
Bachelor's degree	57.9%	738	63.7%	1381	61.5%	2119
Postgraduate degree	15.9%	203	13.7%	297	14.6%	500
**KSA regions**						
< 4 Million	65.9%	839	71.3%	1547	69.3%	2386
1–4 Million	10.4%	132	13.9%	301	12.6%	433
< 1 Million	23.8%	303	14.8%	321	18.1%	524
**Income in Saudi Riyals (SR)**						
Less than SR6000	13.8%	176	19.8%	429	17.6%	605
SR6000–10,000	19.6%	250	25.6%	555	23.4%	805
SR10,001–20,000	42.3%	539	32.8%	711	36.3%	1250
More than SR20,000	24.3%	309	21.9%	474	22.7%	783
**Employment status**						
Government sector	42.8%	545	24.6%	534	31.3%	1079
Private sector	25.6%	326	8.9%	192	15.0%	518
Business	4.2%	54	2.0%	44	2.8%	98
Healthcare practitioner	3.9%	50	2.8%	62	3.3%	112
Student	12.9%	164	26.7%	578	21.6%	742
Unemployed	4.9%	62	31.9%	691	21.9%	753
Retired	5.7%	73	3.1%	67	4.1%	140

### Dental care characteristics of survey participants

About 45% of participants had their last dental treatment within the last six months, while 5% had never visited a dentist. The majority received their last dental care in private clinic (77.8%), with getting a restoration/prosthesis as the most common type of treatment received in the last dental visit (30%). There were no significant differences in characteristics of the last dental visit by gender in this sample (Table 2).

**Table 2:** Last dental visit characteristics of study sample from KSA population (n = 3443)

Characteristic	Male n = 1274		Female n = 2169		Total n = 3443	
	%	n	%	n	%	n
**Time since last dental visit**						
< 6 months	43.0%	548	46.0%	997	44.9%	1545
6 months to 1 year	22.7%	289	21.2%	459	21.7%	748
> 1 year	29.2%	372	28.2%	611	28.6%	983
Never	5.1%	65	4.7%	102	4.9%	167
**Type of dental clinic**						
Public dental clinic	22.6%	288	22.0%	478	22.2%	766
Private dental clinic	77.4%	986	78.0%	1691	77.8%	2677
**Dental treatment received**						
Routine Check-up	13.4%	171	12.0%	260	12.5%	431
Scaling	20.9%	266	18.9%	409	19.6%	675
Restoration/prosthesis	31.9%	406	28.8%	625	30.0%	1031
Root canal treatment	15.0%	191	16.4%	355	15.9%	546
Orthodontic treatment	6.5%	83	11.2%	243	9.5%	326
Extraction	11.1%	142	11.1%	241	11.1%	383
Other	0.6%	7	1.4%	30	1.3%	37

### Emergency, urgent, and routine non-urgent dental care

**Fig. 1:**
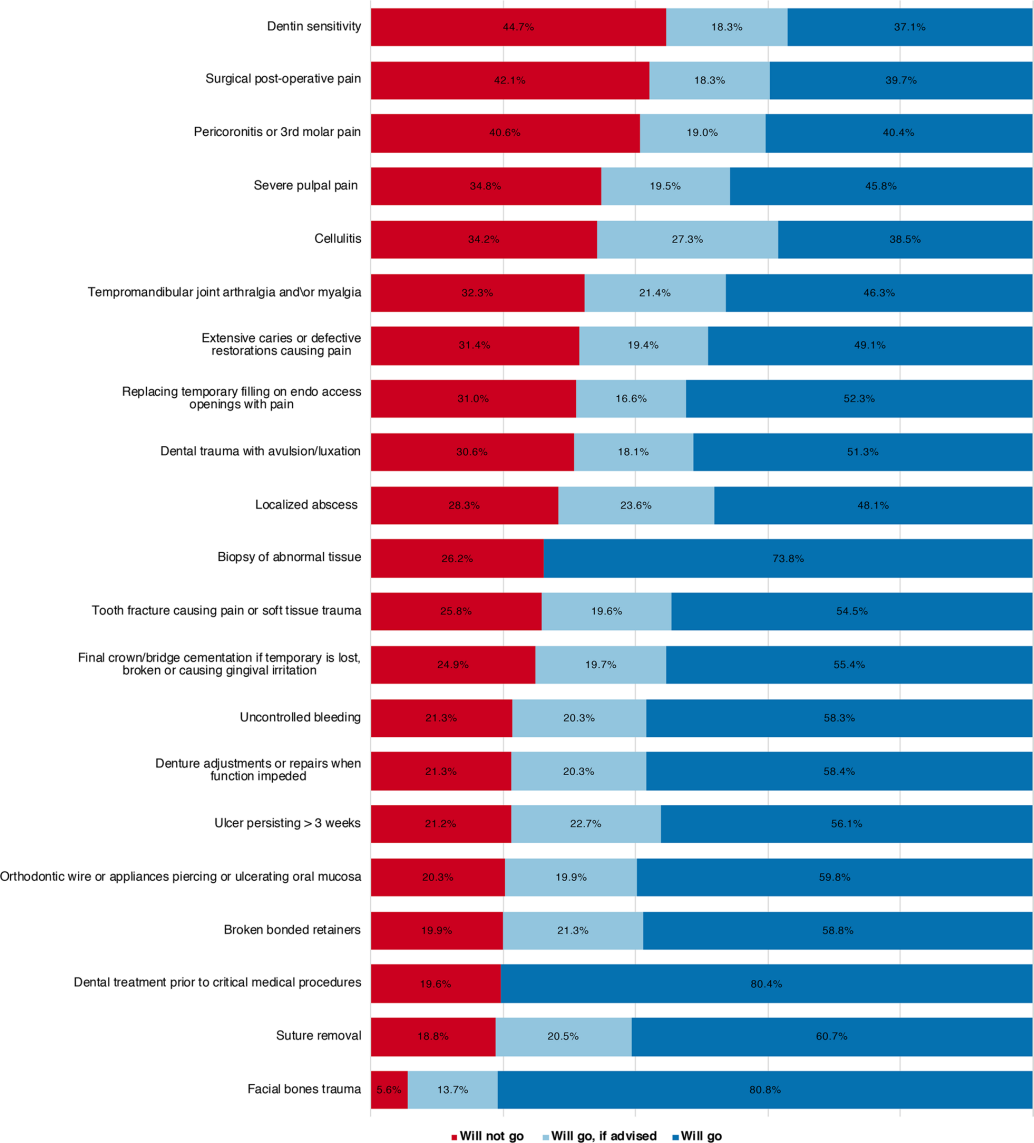
Responses to dental emergencies and urgent dental care situations (n = 3443)

The top 5 emergency and urgent dental care situations participants were willing to go to the dental clinic for were trauma involving facial bones compromising the airway (94.5%), suture removal (81.2%), dental treatment required prior to critical medical procedures (80.4%), broken bonded retainers (80.1%), and poking orthodontic wire (79.7%). On the other hand, the 5 least emergency/urgent dental care situations participants were willing to go were dentin sensitivity (55.3%), surgical postoperative pain (57.9%), pericoronitis or third molar pain (59.4%), pulpal pain (65.2%), and unexpected facial cellulitis that compromises the airway (65.8%) (Fig. 1). On average 20% of respondents reported they would seek teleconsultation as the first step and subsequently going to the dental clinic if advised to do so. However, 15.2% reported they would seek teleconsultation, but would not go to dental clinics even if advised to do so. A number of participants (0.5%) mentioned the use of traditional medicine or herbs in such situations during this pandemic. Another proportion mentioned self-treatment or seeking advice from a pharmacist (0.1–1.2%) should they encounter such conditions during the pandemic.

**Fig. 2:**
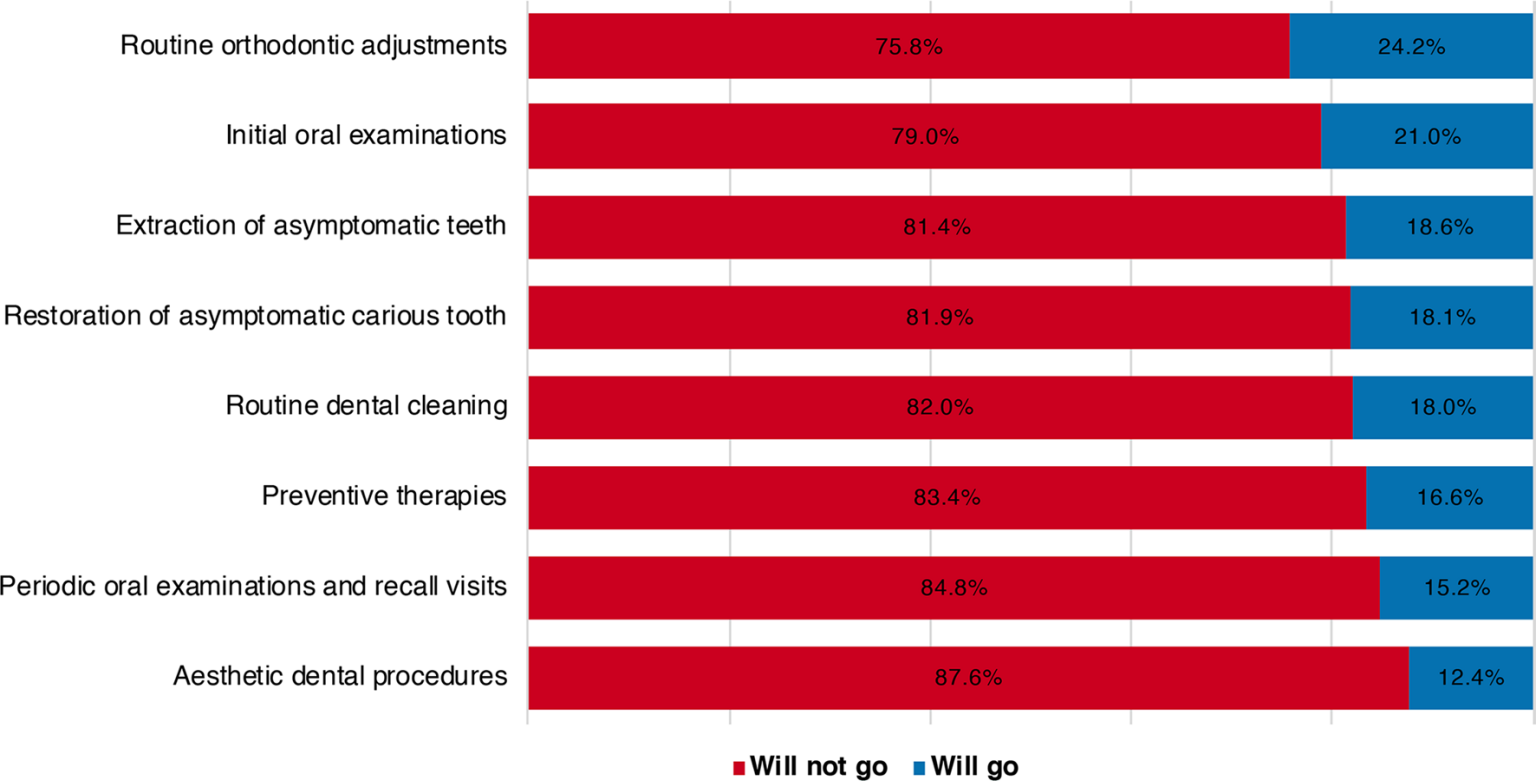
Responses to routine non-urgent types of dental procedures

Participant responses about routine non-urgent type of dental procedures are summarized in Fig. 2. Orthodontic adjustment appointments showed the highest proportion of participants willing to go during the pandemic (24.2%), while esthetic dental procedures showed the lowest proportion (12.4%).

### Factors associated with prediction of participants’ opinion regarding seeking dental care

Table 3 presents multiple logistic regression models predicting the decision between going or not going for treatment of dental emergencies during the COVID-19 pandemic, adjusting for gender, age, education, income, occupation, KSA region, and characteristics of the last dental visit. Females demonstrated significantly higher odds (OR: 1.2, OR 95% CI 1.1, 1.4 to OR:1.6, OR 95% CI 1.3, 1.9, *P* value < 0.001) of choosing not to seek dental care if encountering a dental emergency during the COVID-19 pandemic as in uncontrolled bleeding and cellulitis. Participants who had never visited a dentist also had significantly higher odds of choosing not to go the dentist when experiencing a dental emergency when compared to those who had visited a dentist before (OR: 1.4, OR 95% CI 1.1, 2.1 to OR: 1.9, OR 95% CI 1.1–3.2, *P* value < 0.001). People living in metropolitan regions (> 4 million) were significantly more likely (OR: 1.8, OR 95% CI 1.4, 2.3, *P* value < 0.001) to avoid seeking dental care in the case of uncontrolled bleeding compared to people living in rural regions (< 1 million).

**Table 3:** Predictors for choosing not to go to the dental clinic for dental emergencies during the COVID-19 pandemic among a sample of KSA population (n = 3443)

Dental emergency condition	Model 1	Model 2	Model 3
	Uncontrolled bleeding	Cellulitis that potentially compromise patient’s airway	Trauma involving facial bones, potentially compromising patient’s airway
	Odds ratio (OR) (95% Confidence interval)		
**Gender**			
Male (Reference)			
Female	1.6* (1.3, 1.9)	1.2* (1.1, 1.4)	–
**KSA regions**			
> 4 Million (Reference)			
1–4 Million	0.9 (0.7, 1.1)	–	–
< 1 Million	0.6* (0.4, 0.7)	–	–
**Last dental visit**			
Ever visited a dentist (Reference)			
Never	1.4* (1.1, 2.1)	1.8* (1.3, 2.5)	1.9* (1.1, 3.2)
Model *P* value	*P* < 0.001	*P* < 0.001	*P* = 0.03

^*^P $$\le$$ 0.05

Females exhibited significantly higher odds (OR: 1.3, OR 95% CI 1.1, 1.5 to OR: 2.5, OR 95% CI 2.0, 3.1, *P* value < 0.001) of choosing not to go to the dental clinic for routine dental procedures during this pandemic (Table 4). Participants with an income of more than SR6000 had higher odds of choosing not to go for routine dental procedures compared to those with an income of less than SR6000 (OR: 1.7, OR 95% CI 1.3, 2.2). Respondents having an education level higher than high school also had significantly higher odds of choosing not to go for esthetic procedures compared to those with high school education or less (OR: 1.7, OR 95% CI 1.3, 2.2). People living in rural regions were significantly less likely by up to 50% (OR 95% CI 0.4, 0.6) to choose not going for routine dental care compared to people living in metropolitan regions.

**Table 4:** Predictors for choosing not to go to the dental clinic for routine non-urgent dental procedures during the COVID-19 pandemic lockdown period among a sample of KSA population (n = 3443)

Routine dental procedures	Model 1	Model 2	Model 3
	Routine orthodontic adjustments	Routine dental cleaning	Aesthetic dental procedure
	Odds ratio (OR) (95% Confidence interval)		
**Gender**			
Male (Reference)			
Female	1.3* (1.1, 1.5)	2.1* (1.8, 2.5)	2.5* (2.0, 3.1)
**KSA regions**			
> 4 Million (Reference)			
Million	0.9 (0.7, 1.1)	0.8 (0.6, 1.1)	0.8 (0.6, 1.1)
< 1 Million	0.5* (0.4, 0.6)	0.5* (0.4, 0.6)	0.4* (0.3, 0.5)
**Income in Saudi Riyals (SR)**			
Less than SR6000 (Reference)			
SR6000–10,000	–	1.2 (0.9, 1.5)	1.2 (0.9, 1.6)
SR10,001–20,000	–	1.7* (1.3, 2.2)	1.8* (1.4, 2.4)
More than SR20,000	–	1.7* (1.3, 2.3)	2.0* (1.4, 2.8)
**Education level**			
Highschool or less (Reference)			
Diploma degree	–	–	1.7* (1.1, 2.7)
Bachelor's degree	–	–	1.7* (1.3, 2.2)
Postgraduate degree	–	–	1.3 (0.9, 1.9)
Model *P* value	*P* < 0.001	*P* < 0.001	*P* < 0.001

^*^P $$\le$$ 0.05

### Pain threshold

The median NRS-11 score for the pain threshold that would make participants go to the dental clinic during this pandemic was 7. Females responded with significantly higher pain threshold than males (mean NRS-11 of 7.2, 6.9; respectively). Those who had never visited a dentist also reported a higher threshold than those who had visited a dentist before. Patients getting dental care in private dental clinics had significantly lower threshold than those following up at public dental clinics. Patients living in rural regions of KSA reported significantly lower threshold than those living in more populated regions. Gender, ever versus never visiting a dentist, and KSA regions variables were statistically significant in predicting NPS-11 score (Model *P* < 0.001) (Table 5).

**Table 5:** Pain threshold based on participants’ characteristics among a sample of KSA population (n = 3443)

Characteristics	Mean ± SD	Median	*P* value	B (95% CI)
**Gender**			< 0.001*	
Male	6.9 ± 2.2	7		Reference
Female	7.2 ± 2.1	7		0.4 (0.3, 0.6)*
**Visiting dentist**			0.02*	
Ever	7.1 ± 2.2	7		Reference
Never	7.5 ± 2.3	8		0.3 (− 0.006, 0.7)
**Last dental visit type of clinic**			0.01*	
Public	7.3 ± 2.2	7		Reference
Private	7.1 ± 2.1	7		− 0.2 (− 0.4, − 0.02)*
**KSA regions**			0.002*	
> 4 Million	7.2 ± 2.1	7		Reference
1–4 Million	7.1 ± 2.2	7		− 0.2 (− 0.4, 0.1)
< 1 Million	6.9 ± 2.4	7		− 0.3 (− 0.5, − 0.1)*

B, Standardized coefficient of multiple regression; CI, Confidence Interval

^*^Statistical significance using Mann–Whitney or Kruskal–Wallis tests, as appropriate

## Discussion

The aim of the present study was to examine social determinants associated with decisions to seek dental care in Saudi Arabia during the COVID-19 pandemic. We found that social disparities do exist in emergency dental care seeking decision-making in Saudi Arabia during this pandemic. The participants had some fear regarding risks involved with going to a dental clinic during the pandemic, and therefore a large proportion opted not to go to the dental clinic in many urgent dental situations, but rather preferred teleconsultations and managing the situation at home. There was a small proportion of participants who did not mind going to the dental clinic for routine, non-urgent procedures during the pandemic. When examining the participants’ opinion regarding seeking dental care for dental emergencies during the COVID-19 pandemic, we found that the proportion of people willing to go to the dental clinic varied between 94.4% for trauma of facial bones compromising the airway to 65.8% for cellulitis potentially compromising the airway. It was surprising that only 65.8% were willing to go for dental care in the case of cellulitis, despite showing a photograph of a severe cellulitis condition and mentioning that it was compromising the airway. This may indicate the need to educate the public about life-threatening dental conditions that necessitate emergency dental care. For urgent dental care conditions, the proportion of those who are willing to go the dental clinic ranged from 55.3% to 81.2%. One possible explanation is that people were highly concerned about virus transmission and therefore preferred to manage the dental situation in a way that avoided going to the dental clinic. One interesting finding was that 12.4% to 24.2% were still willing to go for routine non-urgent dental procedures during the lockdown, despite the recommendation to postpone such procedures at that time. Insufficient knowledge among the public about COVID-19 was observed in a recent study in Riyadh, KSA. It showed that only 58% of the public had good knowledge about COVID-19 facts with 81% reporting adequate practice during this pandemic [25].

In the present study, on average 35.2% reported seeking teleconsultation as the first resort if encountering emergency or urgent dental care. During this pandemic, the importance of teleconsultation has risen and is now considered an essential service [26, 27]. Despite the known advantages of teledentistry in improving access to dental care and providing cost-effective service [28], its development was relatively slow. This pandemic increased teledentistry development and utilization drastically [29]. Further benefits of teledentistry have emerged as a result of the COVID-19 pandemic, including ensuring the continuity of care while trying to avoid direct physical contact. Evaluation of patients’ satisfaction with teledentisty during the COVID-19 pandemic was conducted in the UK. East Surrey Hospital patients reported their satisfaction and positive experience with teledentistry [30].

When examining the social determinants associated with the decision to seek emergency dental care during the COVID-19 pandemic in Saudi Arabia, female residents, people living in metropolitan regions, and those who had never gone to the dentist were more likely to choose not to go to dental clinics. Another important finding is that those with income greater than SR6000 (poverty level) and who have a high school diploma or higher education were more likely to choose not to go to the dental clinic for non-urgent routine dental care compared to those with lower income and education levels. This reflects that those with higher socioeconomic status were more concerned about COVID-19 transmission in the dental setting.

Health care-seeking behavior varies by gender, age, socioeconomic status, illness type, access to health services, and perception of the quality of service [31]. It was interesting to note that female participants were more likely to choose not to seek dental care for emergency dental conditions during the COVID-19 pandemic. Our finding is supported by Berglund et al. who found that women, those with lower education level, renters (compared to those owning a private house), and those with financial problems were associated with increased risk of refraining from seeking dental care [32]. In the contrary, Thompson et al. reported that females tend to seek heath care more than males [33]. In this pandemic, media coverage could be a contributing factor influencing an individual’s health-seeking behaviour as the media is heavily involved through many channels [34]. Garfin et al. found that repeated media exposure could lead to increased anxiety and misplaced health-seeking behaviors [35]. Moreover, perceived risk also does affect health-seeking behavior according to Gonzalez-Olmo et al., who reported that those who perceived the dental clinic as a high-risk location for acquiring COVID-19, reported avoiding dental services [36]. In another study, 97% of patients reported the risk of infection during a dental visit as moderate or high, with 80% reporting that the main factor affecting their dental visit decision was being afraid of the potential of transmission in the hospital setting [37].

In our study, the mean pain score that participants chose as the threshold to go to dental clinic during the pandemic was 7.2 ± 2.2. This is in agreement with a Norwegian study that reported a mean pain score of 7.0 ± 2.6 that would make patients seek dental emergency treatment [38]. Women reported higher pain threshold than men in our study. This is in line with findings of previous studies [39].

The study has several strengths. The web-based survey enabled access to people who would be difficult or impossible to reach, especially during the lockdown [40]. Sample size and representativeness of national demographics in several indicators is another strength. The demographic characteristics of our sample were comparable to the Saudi census statistics [41] in terms of age, marital status, income, and KSA regions. The representation of KSA regions in the study sample was reasonable, with participants from regions with more than 4 million population comprising 69.3%, which is comparable to national statistics (66.6%) [42].

This study has several limitations, including the limitation of the snowball sampling strategy and self-selection bias inherent with web-based surveys which may limit the study generalizability. However, rapid online surveys have been shown to be a great tool for capturing the public’s opinion and knowledge especially during pandemics [43]. Another limitation is having a higher proportion of female participants (63%) compared to males, which is higher than national figures (43%). However, Rübsamen et al. findings do support this gender differential responses to web-based surveys [44]. Another limitation is the fact that our study sample may not be a true representative of KSA population as participants had to be literate and active on social media. According to the most recent data, 84.9% of the Saudi population are literate [42] and 58% (equivalent to 18.3 Million) of them are active on social media [45]. Social desirability bias is also a possible limitation; however, it has been found to be of less concern in web-based surveys than in telephone or in-person surveys [46, 47]. Non-response bias is another potential limitation, yet since sociodemographic characteristics of the study sample are not different from national figures it is not a major concern. This study presents new insight regarding the social determinants of health-seeking decisions for seeking emergency, urgent, and routine dental care during the COVID-19 pandemic.

## Conclusion

Social disparities were found in emergency dental care seeking decision-making in Saudi Arabia during the COVID-19 pandemic. It was alarming that some people were afraid to seek dental care for life-threatening dental emergencies as cellulitis during this pandemic. On the other hand, some people were willing to go for non-urgent and cosmetic reasons. This reflects the importance of increasing public health awareness and governmental regulations especially during pandemics. In addition, preference for using teleconsultations did leap during this pandemic. It would be interesting to explore actual dental care utilization during the COVID-19 pandemic in Saudi Arabia in future research.

## Supplementary Information

**Additional file 1:** Survey.

## Data Availability

The datasets generated and/or analysed during the current study are not publicly available due to the institution regulations, but are available from the corresponding author on reasonable request.

## Abbreviations

COVID-2019: Coronavirus disease 2019
KSA: Kingdom of Saudi Arabia
MOH: Ministry of Health
CDC: United States Centers for Disease Control and Prevention
ADA: American Dental Association
UK: United Kingdom
NRS: Numeric Rating Scale
SR: Saudi Riyals
SARS: Severe Acute Respiratory Syndrome
MERS: Middle East Respiratory Syndrome
TMJ: Temporomandibular Joint
