# Supplementary material for: Social determinants of seeking emergency and routine dental care in Saudi Arabia during the COVID-19 pandemic
Source: BMC Oral Health. 2021 Apr 26;21:212. doi: 10.1186/s12903-021-01577-1 (PMC8074701; doi:10.1186/s12903-021-01577-1)
Supplement: Supplementary file 1 — Additional file 1: Survey. [file 12903_2021_1577_MOESM1_ESM.docx]

**Additional file 1:** Survey

| **Nationality:**   - Saudi - Non-Saudi   **Gender:**   - Female - Male   **Age:**   - 18-24 years - 25-29 years - 30-39 years - 40-49 years - 50-59 years - 60 years and older   **Marital status:**   - Single - Married - Divorced - Widowed   **Region:**   - Mecca region - Medina region - Riyadh region - Al-Qassim region - Eastern province region - Asir region - Tabuk region - Ha'il region - Northern borders region - Jazan region - Najran region - Al Bahah region - Al Jawf region   **What is your highest level of education?**   - Highschool or less - Diploma degree - Bachelor's degree - Master - PhD/Board | **Occupation:**   - Government sector employee - Private sector employee - Own business/freelancer - Healthcare practitioner - Student - Housewife - Unemployed - Retired - Other __________   **Income per month in SR**   - Less than 6,000 - 6,001-10,000 - 10,001-20,000 - 20,001-50,000 - 50,001-10,0000 - More than 100,000   **When was your last dental visit?**   - Less than 6 months - 6 months to a year ago - More than a year ago - Never visited a dentist   **Where was your last dental treatment?**   - Public dental clinics - Private dental clinics   **What was the type of treatment you received in**  **your last dental visit?**   - Routine Check-up - Scaling - Restoration - Prosthesis - Root canal treatment - Orthodontic treatment - Extraction - Other __________ |
| --- | --- |

| **We are going to list some dental conditions and we want to know what you would do if you experienced any of them NOW during the COVID-19 outbreak?**   \|  \| **Go straight to ER or dental clinic** \| **Teleconsultation (calling 937 or dentist) and will go to dental clinic if advised** \| **Telconsultation (calling 937 or dentist) and will not go to dental clinic even if advised** \| **Other: specify** \| \| --- \| --- \| --- \| --- \| --- \| \| Uncontrolled bleeding in your mouth. \|  \|  \|  \|  \| \| Swelling in the face (cellulitis) narrowing the airway due to tooth infection. \|  \|  \|  \|  \| \| Trauma involving facial bones fractures compromising airway because of accident or falling. \|  \|  \|  \|  \| \| Severe dental pain from pulpal inflammation. \|  \|  \|  \|  \| \| Pain from wisdom tooth or the gum surrounding it. \|  \|  \|  \|  \| \| Severe pain after a dental surgical procedure or extraction. \|  \|  \|  \|  \| \| Abscess or localized bacterial infection resulting in localized pain and swelling. \|  \|  \|  \|  \| \| Tooth fracture resulting in pain or causing trauma to surrounding soft tissue (e.g. tongue and cheek). \|  \|  \|  \|  \| \| Dental trauma causing the tooth to completely fall out or become loose. \|  \|  \|  \|  \| \| Dental treatment required before critical medical procedures (e.g. cancer therapy). \|  \|  \|  \|  \| \| Temporary crown is lost, broken, or causing gum irritation and need to be replaced with the final crown. \|  \|  \|  \|  \| \| Extensive dental cavities or defective restorations causing pain. \|  \|  \|  \|  \| \| To remove surgical suture. \|  \|  \|  \|  \| \| To adjust or repair your removable denture when function is impeded (eating and talking). \|  \|  \|  \|  \| \| To repair fallen temporary restoration causing pain. \|  \|  \|  \|  \| \| To snip or adjust your orthodontic wire or appliances that is piercing or ulcerating your mouth. \|  \|  \|  \|  \| \| Have ulcer in mouth that is persisting for more than 3 weeks. \|  \|  \|  \|  \| \| Painful sensitivity in your teeth. \|  \|  \|  \|  \| \| Pain in your jaw joints or jaw muscles preventing you from sleeping. \|  \|  \|  \|  \| \| Fixed retainer that broke causing pain and you couldn't deal with it at home. \|  \|  \|  \|  \| \| Biopsy of abnormal tissues in the mouth. \|  \|  \|  \|  \|   **Please answer the following questions based on your opinion, whether you would go or not to the dental clinic NOW during COVID-19 outbreak for those dental**  **procedures?**  I will go to the dental clinic for periodic oral examinations and recall visits.   - Yes - No   I will go to the dental clinic for the initial oral examination visit.   - Yes - No   I will go to the dental clinic for a routine dental cleaning.   - Yes - No   I will go to the dental clinic for preventive therapies (Fluoride application and fissure sealants).   - Yes - No   I will go to the dental clinic for regular braces adjustment (orthodontic) appointments.   - Yes - No   I will go to the dental clinic to extract a tooth that doesn't cause pain.   - Yes - No   I will go to the dental clinic to restore simple decay that is not causing pain.   - Yes - No   I will go to the dental clinic for aesthetic dental procedures (as bleaching, veneers).   - Yes - No   **In a scale of 0-10, where 0 means no pain and 10 unbearable pain, what is the pain level that you wouldn't withstand and will make you go seek dental care during**  **the COVID-19 outbreak?**  0 1 2 3 4 5 6 7 8 9 10 |
| --- | --- | --- | --- | --- | --- | --- | --- | --- | --- | --- | --- | --- | --- | --- | --- | --- | --- | --- | --- | --- | --- | --- | --- | --- | --- | --- | --- | --- | --- | --- | --- | --- | --- | --- | --- | --- | --- | --- | --- | --- | --- | --- | --- | --- | --- | --- | --- | --- | --- | --- | --- | --- | --- | --- | --- | --- | --- | --- | --- | --- | --- | --- | --- | --- | --- | --- | --- | --- | --- | --- | --- | --- | --- | --- | --- | --- | --- | --- | --- | --- | --- | --- | --- | --- | --- | --- | --- | --- | --- | --- | --- | --- | --- | --- | --- | --- | --- | --- | --- | --- | --- | --- | --- | --- | --- | --- | --- | --- | --- | --- |
